# The design of an upper arm prosthesis utilising 3D printing conceived for the 2020 Tokyo paralympic games: A technical note

**DOI:** 10.1177/20556683221113309

**Published:** 2022-07-05

**Authors:** Bryce Dyer, Richard Glithro, Abigail Batley

**Affiliations:** Department of Design & Engineering, 6657 Faculty of Science & Technology, Bournemouth University, Poole, UK

**Keywords:** Cycling, prosthesis, amputee, athlete, 3D printing

## Abstract

This article describes the design and development of an upper-limb prosthesis for a current elite level paracyclist that was conceived for use in the 2020 Paralympic Games. The prosthetic limb was intended specifically for use in cycling time trial events. These are held on the road and in the velodrome whereby the athlete rides using a tucked aerodynamic position. The prosthesis was developed using computer aided design software and an extensive use of the 3D printed manufacturing process. The resulting technical note illustrates the design methodology and manufacturing considerations for a high performance form of assistive technology. However, it results in a solution that challenges the traditional aesthetic of prosthetic limbs intended for sport and physical activity.

## Introduction

To undertake cycling whilst possessing any level of lower-limb amputation may often require some form of specialised prostheses to be developed.^1^ This technology provides a unique visible distinction between those who possess limb absence and those who do not because the form of a prosthetic limb can be manipulated to suit the nature of the sporting event it is intended to support.^2^ With respect to the sport of paracycling, much of this design is primarily influenced by the biomechanical requirements of the event and the reduction of aerodynamic drag.^2^

Prosthetic limbs are currently allowed for use by athletes when competing in cycling with a disability. A formalised framework for sports assistive technology development was proposed by Dyer^1^ and outlined a multi-disciplinary approach. However, it is conceded that more case studies were required to support its use. Part of the Dyer approach required the detailed review of any governing body legislature, the identification of their limits and the exploitation of any opportunities.^1^ In the case of paracycling, governance is overseen by the Union Cycliste Internationale (UCI). The UCI’s rules regarding prostheses is predominantly outlined by rule 16.14.002 and is as follows.

*16.14.002A All requests of homologation for prostheses, orthoses or impairment adaptations to any cycle must be submitted in writing with proper explanation and pictures to UCI for approval, in accordance with the procedure established by the UCI available on its website. Such request must be received at the UCI at least 3 months before any event that the athlete review (R) or confirmed (C) wants to participate in. New athletes (N) must submit such request 1 month before the date of the event must be provided in the request. In case the adaptation is approved, approval number (self-adhesive) and a certificate will be sent to the athlete to present at any event. All adaptations, prostheses or orthoses must be approved by the UCI prior to the event. Athletes are not allowed to race without an approved device.*

### 16.14.003 In no case may an energy storage or assistance mechanism be integrated into an bis orthopaedic brace/prosthesis

These rules demonstrate a level of ambiguity because the dimensions, shape, form, colour, materials and method of manufacture of a prosthetic limb are not specified in any way. The only functional limitation is that no energy assistance mechanisms can be used. However, from a design perspective, this is problematic as the rule suggests that the governing body would determine the acceptance of any new assistive technology subjectively and without then being aware of the full extent of its capabilities or design rationale. Furthermore, the ability to veto the use of a prosthetic limb at a point when it has already been manufactured would be seen as costly, wasteful and potentially upsetting to its end-user. However certain precedents already exist within the sport of paracycling with several aerodynamically shaped lower-limb prosthesis designs already being witnessed in service.^2,3^

### 3D printing of prosthetic limbs

Most sports prosthetics are highly bespoke in their design and manufactured in clinic-based workshops. This can be a lengthy process as the physical and biomechanical demands required by the end-user and the sport they participate in will vary on a case-by-case basis.^4,5^ By utilising advances in 3D printing, scanning and materials, the time scale of designing and producing sports prosthetics can be dramatically reduced.^4^ This process ultimately allows for bespoke devices to be designed in an interactive and collaborative process between the end-user and the designer.^5^

3D printing or ‘additive manufacture’ is the ability to create forms that have been designed within a virtual workspace and are then realised via machines which replicates the artefact physically by depositing subsequent layers of polymers or a range of other materials. 3D printing as a process has previously been utilised to create components such as; lower limb prosthetic fairings, lower limb sockets and upper limb prostheses.^6^ Some examples of sports prostheses that have been 3D printed in the past include a cycling prosthesis for Denise Schindler to compete with at the 2016 Rio Paralympic Games^4^ and an upper limb fencing prosthetic created for academic research.^5^ Denise Schindler worked with Autodesk to become the first cyclist to compete with a 3D printed prosthetic leg at the Paralympic Games.^4^ The process in such case studies was much faster than the traditional process of an orthopaedic technician taking a plaster cast of the residual limb and then hand producing a prosthetic. Such processes can be both long and expensive.^5^

The current prosthetic limbs that have been 3D printed have utilised materials such as polycarbonate and polylactic acid, which are low cost, low quality but don’t have many desirable engineering material properties.^4,5^ However, the relatively recent increased material feasibility for 3D printing would mean more structural designs could be created.^5^ Composite 3D Printing offers a resolution to these limitations by printing a material which possesses short reinforcement fibres mixed with a thermoplastic matrix or continuous composite printing. Fused deposition modelling (FDM) composite printing allows for reinforcements to be accurately placed and structures to be optimised per layer, allowing an increase in design freedom and mechanical performance.^7^ Current industries using 3D composite printing include aerospace and Formula 1. Current applications include jigs and fixtures, moulds and end use components.^8,9^

Ultimately, the blending of 3D printing and functional composites will enable new applications for the medical and sports industries^7,10^ which could facilitate the next generation of 3D printed medical devices, including prosthetic limbs.^6,10^

## Design philosophy

The design brief for this project was to create an arm-based prosthetic limb intended for elite-level para-cycling competition using modern 3D printing development. The Dyer framework (2018) was utilised for this project to identify the key objectives from such a device. Following this, a formal specification was derived that identified the key performance issues unique to the end-users sport. The summarised main design criteria were:

• A reduction in aerodynamic drag^3^ with the end-user positioned when using their intended racing bicycle.
• For the prosthetic limb to possess an aspect ratio maximum that was no greater than existing legislated bicycle componentry to legitimise any concerns over a component offering an unfair advantage.^2^ This aspect ratio maximum was set as 3:1 which was in line with some historical legislation of bicycle component design and the precedents of lower-limb prosthesis that had already been in service.^2,3^
• A form that allows for some intolerance or movement by it when used at race-pace exercise intensity and assuming some degree of asymmetry.^2^
• The ability to create prototypes in a relatively short timescale to meet the competitive needs of both athlete and their coaches.^4^
• The end-user wanted to utilise their older prosthetic limbs when in training. As a result, the method of attachment from athlete to bicycle would not be re-designed so was not considered within the scope of this project.

A single participant was utilised for this project. They would be used to offer feedback on the concept designs, trial any developmental models and serve as the eventual end-user of the prosthesis. This participant was a male paracyclist who competed at an international level and was still competitively active. The participant gave their informed consent and this project received institutional ethics approval from the authors host institution (No: 10012).

### Design process

The initial design concepts were modelled virtually utilising a combination of SolidWorks and Autodesk 3D Studio Max computer software. The participant would also have their residual limb and torso regions scanned to ensure that any design would fit within this area without inhibiting their physical act of cycling or when starting their event from a static start gate when racing on a velodrome. A NACA 0012 aerofoil served as the basis of the aerodynamic form being used for the design.^11^ This aerofoil was also used in previous case studies.^2,3^ The NACA aerofoil overall size was defined by measuring the participants current socket and using that measurement to determine the foils width. A previous case study proposed that the most effective design from its limited concepts was that of a 6:1 aspect ratio. This had been based upon several cross sectional forms established from contemporary bicycle frame design. A Kamm tail was then applied to this foil at the point where the aerofoils tail width was 50% of the maximum width of the overall foil. Kamm tails have been demonstrated to be laterally and torsionally stiff as well as possess similar aerodynamic properties to that of a full foil shape.^12^ Furthermore, use of a Kamm tail design was chosen to reduce the aerodynamic drag caused by some minor rotational movement of the amputees limb witnessed in other case studies,^3^ to achieve the deepest theoretical aerofoil possible and finally to comply with the maximum 3:1 aspect ratio limitation of this project. Due to this project being that of an arm rather than a leg, the 3D model of this had relief added in several areas where the computer software identified where the athlete would strike the prosthesis whilst cycling. This need was undertaken initially using the computer aided design software and then validated by the athlete when exercising at the intended race pace. This process was as per previous recommendations.^1^

It should also be noted that a range of concept variants based upon this design were created by having change parts that were affixed this design by using magnets. This method was a cost, time and resource effective method of generating derivative designs although most of these were not progressed from their concept stage.

The method of attachment of the prosthetic to the participant utilised a standard silicon liner over the stump with a socket and valve as per conventional prosthetic limb use. As per the projects stated design criteria, the prostheses to bike attachment was not redesigned and this method of attachment involved a ball and socket joint. The socket was mounted to the bicycles handlebars where the participants elbow would conventionally be expected to be when riding a bike in an aerodynamic position. The ball spigot would then be mounted at the distal end of the prosthesis. This solution allowed the participant to clip in easily to the bicycle and still retain the use of their older prostheses when training. The ball and socket connection provided a broad range of degrees of freedom so that any upper body movement by the athlete could be accommodated without the prosthesis detaching from the bicycle. In summary, this meant that a biological hand retained control of the bicycle on one side with the prosthetic and its connector on the other, thereby providing a reasonable level of bi-lateral contact points.

The designers established a core design philosophy that the rider, their bicycle and the prosthetic limb itself would be aerodynamically measured as one overall form rather than the prosthesis itself being considered as an independent and isolated product. The reason for this was based upon the interactions that one component may have on another when assessing aerodynamic drag.^13^

### 3D printing manufacture

Once a final design had been determined, the method of 3D printing and additive manufacturing then took place. A first proof of concept model was manufactured from ABS polymer using a Stratasys Fortus 360mc 3D printing machine (Warwickshire, UK). This model was intended for an initial test fit for feedback from both the athlete and their coaches.

**Figure 1.**
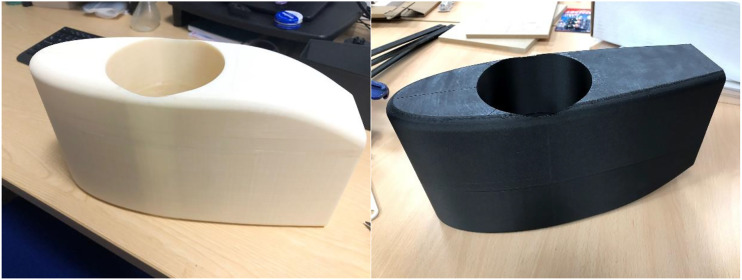
Arm aerofoil concept models in polymer (left) and composite (right).

The same concept model was also produced on a Markforged Mark Two 3D printing machine (Minnesota, US), from onyx matrix with carbon fibre reinforcement. These full sized 3D printed concepts are shown in Figure 1.

The white model in Figure 1 was 3D printed in a polymer material whereas the black model is the same design but 3D printed in a composite material. Due to the size limitations of the second iteration of the design (Figure 1, right image), this model was also printed in two halves and then bonded together. This lighter model was intended for functional testing when the athlete was cycling. Both model forms would then be slid over the top of the athletes existing socket to assess for any strikes from the legs or torso when the athlete was cycling.

**Figure 2.**
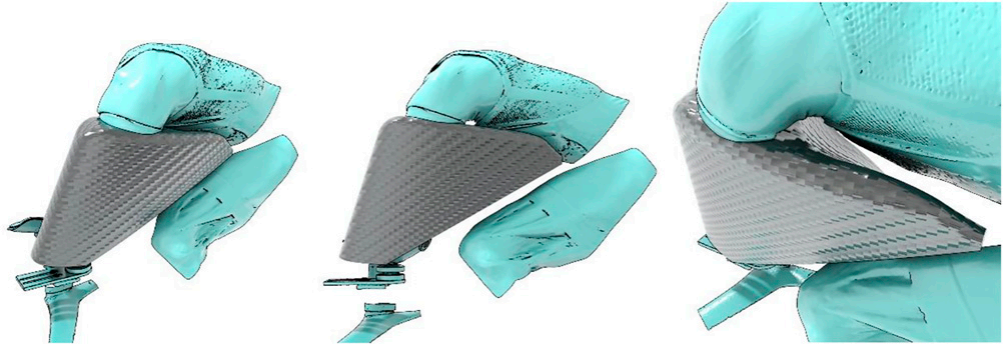
Views of final prosthesis design (in grey) and computerised scan of rider leg and torso (in turquoise).

Feedback from the proof of concept model demonstrated areas that required further development. This required relief areas added in order for the athlete to have a full range of motion without striking the prosthetic limb. This was assessed in reality by the participant riding on their race bike but also by scanning the athlete and then test fitting the prostheses in a virtual environment. Having done this, the two key areas that required relief were around the athlete’s ribcage and knee joint at maximum hip flexion (where the knee is closest to the ribcage). The images showing various views of the final design when attached to the athlete in the virtual environment are shown in Figure 2.

Figure 2 shows the computer aided design of the prosthesis aerofoil when worn by the athlete. The turquoise areas are digitised body scans of the torso and upper leg area of the athlete. These body scans aided the designers ascertain the best fit between prosthesis and athlete. The relief areas (third image from right) show how the prosthesis fits against the rider when in their aerodynamic riding position.

Two evolved final designs were ultimately produced. The first was identical in form to the design illustrated in Figure 2. The second design comprised a more conservative aesthetic that was shorter in its overall length. This conservative option was created as it was felt that the first design could be seen as controversial in comparison to contemporary prosthesis designs whereas the second was subjectively felt to be at the perceived limit that would be deemed visually acceptable to the sport’s governing body and officials.

**Figure 3.**
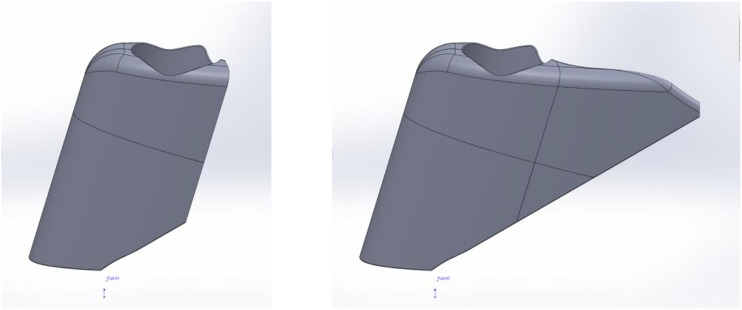
Advanced concept computer aided designs (left and right) with magnetically attached tail version (right).

In order to reduce costs and print time, a multi part model was created that allowed for both of the different designs to be tested whilst minimising print time and cost. This was achieved by having additional parts that would attach via magnets. These were added to the model after printing and located in debossed areas of the attachment face. These models were produced from ABS polymer using a Stratasys Fortus 360mc 3D printing machine and are shown in Figure 3.

**Figure 4.**
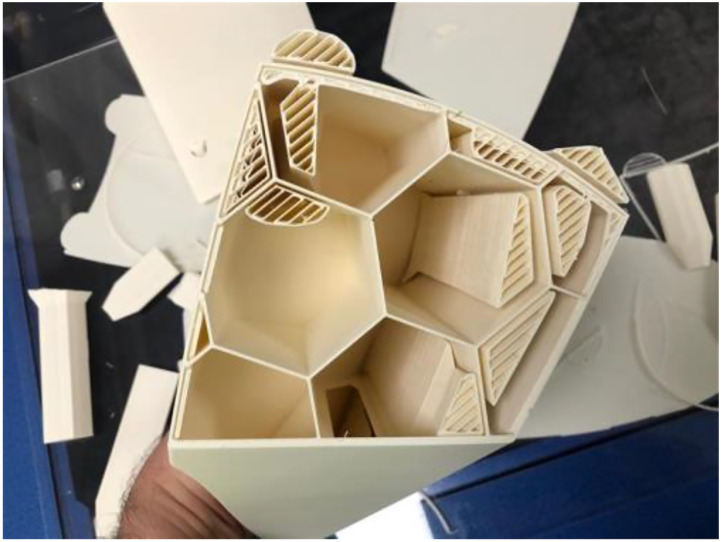
Internal manufacturing detail of prosthesis model.

A multipart printing technique was used to allow for the removal of support material. This led to the production of two prostheses being printed. Each one was made up of two parts. Each half possessed a lip and groove feature to its open face edge to ensure both halves were aligned perfectly. The two halves would then be bonded together. The internal structure of the main parts also show honeycomb-like ribs which purely serve to provide additional structural rigidity to the model and to allow for a thin outer wall to be used to reduce the overall weight of the prototype. This internal reinforcement of the prosthesis model is shown in Figure 4.

**Figure 5.**
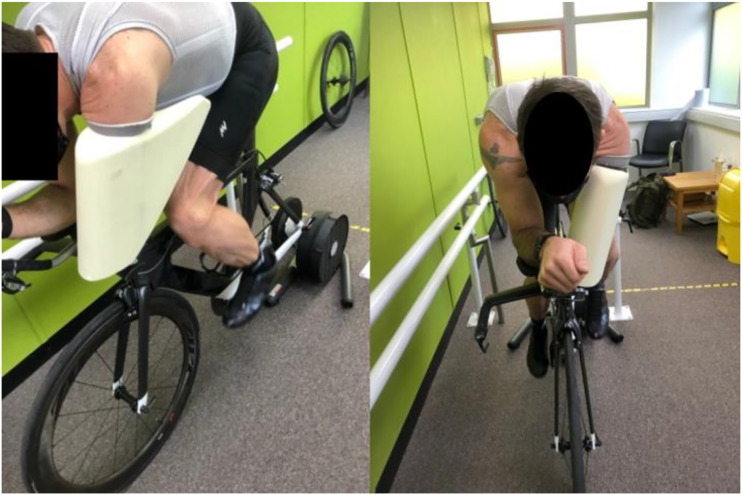
Initial test fit of final prosthesis design to the athlete.

Final revisions were made to the 3D printed model to make it suitable to CNC machine the foam core for the final composite version that would be manufactured by a prosthetist. This included removal of the central hole and patching of both ends to match the existing contours of the design. This would create a one-piece prosthetic limb comprising the prosthetic shell outlined in this paper and the socket and liner manufactured by a prosthetist. Upon successful final testing by the athlete, the design was then transferred to the athletes’ prosthetist for completion. The prosthetist then manufactured the final prosthesis using a carbon fibre composite material outer lay-up over a foam core. The test fitting of the prosthesis to the athlete prior to its carbon fibre lay-up is shown in Figure 5.

## Discussion

The eventual prosthetic limb was manufactured and completed on time. However, it is stated that the resulting prostheses design could not be utilised at the Tokyo Paralympics themselves. This was because the end-user was unable to obtain formal approval of the design due to a lack of competitive events held prior to the games due to the Covid-19 global pandemic. This prevented the full governing body approval process of rule 16.14.002 which was detailed earlier in this paper. Nonetheless, this process could be attempted for competitive use in the future.

The recommended future development for this design would include wind tunnel testing of both the device and the athlete to validate its specific gain in performance over any previous prosthesis the athlete had used. Furthermore, the design itself was intended to see further iteration by adding more material to the top face of the foil to blend it directly into the shoulder region of the specific athlete. This would visually integrate the form of the prosthetic limb with that of the human body and potentially reduce the aerodynamic drag further by smoothing the air flow as it transits to, over and round the athlete.

This design did not have any activity given to its aesthetic surface finish or appeal. It has been indicated in recent studies that athletes with limb absence see desire and value in the customisation of their assistive technology – even in a competitive or scientific environment.^14^ This can include colouring and illustrative decoration. It would be recommended that consultation with the athlete would take place to facilitate this once the manufacture of a prosthetic limb has been completed.

The limitations of the 3D printing manufacturing process was the comparably large size of the concept models coupled with the part size restrictions that the Markforged 3D printer could reasonably accommodate. Whilst this potential limitation could be overcome with a larger 3D printer, this may not be feasible for all practitioners, prosthetists or manufacturing facilities. It should also be noted that the removal of the support structures from the 3D prints and post production cleaning up of the prints provided additional time constraints that may not be obvious to those unfamiliar with the 3D printing manufacture process. Such activities need to be accommodated within any project timescales utilising this technology. Finally, the addition of the magnets used to attach additional parts also proved to be challenging due to the need for any part-to-part fit to be flush and to minimise any gap between them. Such gaps could have created unwanted aerodynamic turbulence. Furthermore, considering that this was the last step in the manufacturing process after printing, if the part had been damaged during the insertion of these magnets, it would most likely have led to the need to manufacture fresh parts which would prove costly in terms of time and resources.

Ultimately, it is conceded that there could be concerns from governing bodies that the resulting prosthesis design negatively challenges the conventional aesthetic of prosthetic limbs intended for competitive sport or could be perceived to provide an unfair advantage. For example, the design in this paper does not physically resemble contemporary prosthetic limbs used in either sport or everyday society and arguably creates further apparent ‘cyborgification’ of athletes with a disability. Such concerns have been raised before with respect to the runner Oscar Pistorius^15^ and the use of wheelchairs or prosthetic limbs by other athletes.^16^ However, prostheses of a similar aerofoil shape are already in service for lower-limb absence^17^ and the design proposed in this paper adopted a measurable design envelope to restrict its form. As a result, it is felt that the precedent has already been established and furthermore complies within the rules as they have currently been defined. If any discomfort does result from such prosthetic limb designs, it is argued that formal debate should take place on such ethical issues and that the legislature is then corrected to reflect such outcomes.

## Conclusion

This technical note described the design and development of an upper limb prosthesis for a current elite-level paracyclist that was conceived for use in the Tokyo Paralympic Games held in 2021. The prosthesis was successfully developed and prototyped using computer aided design software and manufactured using 3D printers before resulting in a traditional fabrication process by prosthetists. The resulting design provided the means for a cost effective, high performance solution but challenges the conventional aesthetic of prosthetic limbs intended for competitive paracycling.
